# Risk Factors for Benign Convulsions With Mild Gastroenteritis

**DOI:** 10.3389/fped.2022.925896

**Published:** 2022-06-29

**Authors:** Cuiyun Fang, Wei Fan, Chunsheng Zhang, Yi Yang

**Affiliations:** ^1^Department of Nursing, Liyang People's Hospital, Liyang, China; ^2^Department of Pediatrics, Liyang People's Hospital, Liyang, China

**Keywords:** benign convulsions, gastroenteritis, risk factors, children, rotavirus

## Abstract

**Objective:**

To investigate the risk factors for benign convulsions with mild gastroenteritis (CwG) and provide a basis for the early identification and diagnosis.

**Method:**

We selected children with CwG and acute gastroenteritis in the same period who attended the pediatric department of the Liyang People's Hospital from June 2018 to June 2021. A retrospective analysis of the general data, clinical characteristics, and laboratory test results of the two groups was performed. Finally, we conducted a multifactorial logistic regression analysis to derive the risk factors for CwG.

**Results:**

A total of 82 children were included in the CwG and 93 children were included in the acute gastroenteritis group. Univariate analysis showed no significant differences in gender, age, vomiting, diarrhea, neutrophil, lymphocyte, hemoglobin, platelets, and serum calcium. Statistically significant differences were found in onset season, rotavirus, white blood cells, aspartate aminotransferase, alanine transaminase, serum sodium, and uric acid. Finally, multifactorial logistic regression analysis showed rotavirus (OR, 3.042, 95% CI, 1.116–8.289, *p* = 0.030), serum sodium (OR, 0.811, 95% CI, 0.684–0.962, *p* = 0.016) and uric acid (OR, 1.018, 95% CI, 1.012–1.024, *p* = 0.000) to be independent risk factors.

**Conclusion:**

Characteristics of gastroenteritis symptoms such as vomiting and diarrhea cannot be used to predict the onset of CwG. Rotavirus is an independent risk factor for CwG, and decreased serum sodium and increased uric acid can be used as early warning indicators for CwG.

## Introduction

Benign convulsions with mild gastroenteritis (CwG) are defined as afebrile seizures in children with mild gastroenteritis in the absence of severe dehydration, electrolyte disturbances, or hypoglycemia. CwG has been reported around the world, mostly in East Asia, including Japan, Korea, and China (1). In recent years, a single-center study found that the proportion of CwG among patients hospitalized for initial convulsions has risen from 8.47% to 12.5% (2).

Despite the good prognosis of CwG (3), convulsions in the acute phase are often clustered seizures, which can easily lead to anxiety in the family. Presently, the etiology and pathogenesis of the disease are unclear, and there are no specific diagnostic criteria. The diagnosis is mainly based on the symptoms of gastroenteritis and the specific clinical features of convulsions, while other diseases causing convulsions need to be excluded. Coupled with the difficulty of early diagnosis, the problem of over-examination and excessive anticonvulsant treatment is prominent in clinical practice. Misdiagnosis and mistreatment also exist (4).

The clinical features and prognosis of CwG have been exhaustively reported in previous studies (5–7), but only a very few studies have discussed the risk factors of CwG. Are there differences in the characteristics of gastroenteritis symptoms between mild acute gastroenteritis and CwG? Are there specific factors that can help pediatric clinicians warn of the occurrence of CwG? This study used a multifactorial research approach to investigate independent risk factors for CwG, with the expectation that it may provide a basis and warning indicators for the early diagnosis of CwG.

## Materials and Methods

### Study Population

The study population was obtained from children attending the pediatric department of the Liyang People's Hospital, which is a tertiary care hospital in Changzhou, China. These patients were diagnosed with CwG and acute gastroenteritis from June 2018 to June 2021. This study was approved by the Ethics Committee of Liyang People's Hospital (approval no. 2021057).

### Study Design

We retrospectively analyzed clinical data of children with CwG and acute gastroenteritis through an electronic medical record system. Collected data included general information (gender, age), clinical features (vomiting, diarrhea), and laboratory findings (routine blood tests, serum sodium, serum calcium, hemoglobin, platelets, aspartate aminotransferase, alanine transaminase, uric acid, and rotavirus). In addition, CWG patients' cranial computed tomography (CT) and electroencephalography (EEG) were collected. Cerebrospinal fluid results were also collected in some CWG patients with suspected central nervous system (CNS) infection.

Inclusion criteria for the CwG group: meeting the diagnostic criteria of CwG (8); excluding cases of convulsions with fever, moderate-to-severe electrolyte disorders, and encephalitis; excluding cases with CNS infection, encephalopathy, and neurologic diseases; excluding cases with birth asphyxia, intellectual disability, and abnormal brain development. Inclusion criteria for the mild acute gastroenteritis (AGE) group: meeting the diagnostic criteria of acute gastroenteritis (9); excluding cases of electrolyte disorders and moderate or severe dehydration; excluding cases with a disease duration of more than 7 days; excluding cases with incomplete data.

The collected cases were screened and grouped according to the inclusion criteria of the CwG and AGE groups, and then a case-control study was performed. To derive independent risk factors, univariate and multifactorial analyses of factors that may affect the outcome were performed.

### Statistical Analysis

Categorical variables were statistically described using rates, and statistical inferences were made using the chi-square test or Fisher's exact test. Continuous variables were statistically described by means and standard deviations (SD), and the comparison of means between two groups was performed by Student's *t*-test or Mann-Whitney U-test. Multivariate logistic regression analyses were performed and expressed as dominance ratios (OR) and 95% confidence intervals (95% CI). Multivariate logistic regression analyses were used to examine statistically significant variables in univariate analyses. For all tests, a 2-sided *P* < 0.05 was considered to indicate statistical significance. All analyses were performed using SPSS software (version 26.0; SPSS, Chicago, IL, USA).

## Results

### General Information

A total of 82 children were included in the CwG group, including 37 males and 45 females. The minimum age of onset was 8 months and the maximum age was 48 months, with a mean age of 20.8 ± 7.6 months. A total of 93 children were included in the AGE group, 55 males and 38 females. The minimum age of onset was 1 month and the maximum age was 60 months, with an average age of 19.47 ± 14.36 months. The results of the comparison of the general information of the two groups of cases are shown in Table 1.

**Table 1:** Comparison of general information for CwG and AGE group.

	**CwG**	**AGE**	**Value**	***P***
Sex			3.434	0.064
Male	37(45.1%)	55(59.1%)		
Female	45(54.9%)	38(40.9%)		
Age	20.80 ± 7.63	19.47 ± 14.36	−0.779	0.438

*Data are N (%) or mean ± SD values*.

### Characteristics of Convulsions in CwG

All CwG patients had cranial CT and EEG examinations. No abnormalities were seen on cranial CT in all patients. Eight children revealed a slow background without epileptic abnormality. Six children underwent lumbar puncture testing and showed normal results on cerebrospinal fluid examination. Among the 82 children with CwG, 52 (63.4%) had single convulsive episodes and 30 (36.6%) had cluster episodes. 77 (93.9%) children had convulsions of duration within 5 min, 5-10 min for 5 (6.1%). As for the type of convulsions, 75 (91.5%) were generalized tonic-clonic seizures and 7 (8.5%) were focal tonic-clonic seizures. The interval between convulsive episodes and the onset of gastroenteritis was 23 (28.0%) for 0–24 h, 39 (47.6%) for 24–48 h, 17 (20.7%) for 48–72 h, and 3 (3.7%) for ≥72 h. A total of 22 children were treated with anticonvulsant drugs, which were first applied by an enema with 10% chloral hydrate (0.5 ml/kg). In four of the 22 children who received an initial anticonvulsant drug, the convulsions did not resolve after 5 min and were relieved by the administration of phenobarbital (5 mg/kg) intramuscularly. After a 6-*month* to 2-*year* follow-up, *three* cases of CwG recurred, *four* cases experienced febrile convulsions, and *non*e of the patients acquired epilepsy.

## The Univariate Analysis

As shown in Table 2, univariate analysis showed statistical differences in onset season, rotavirus, white blood cells, aspartate aminotransferase, alanine aminotransferase, serum sodium, and uric acid. Vomiting, diarrhea, neutrophil, lymphocyte, hemoglobin, platelets, and serum calcium did not differ significantly.

**Table 2:** Univariate analysis between the CwG group and the AGE group.

	**CwG(*n* = 82)**	**AGE(*n* = 93)**	**Value**	***P***
Onset season			28.867	0.000^*^
Spring	16(19.5%)	17(18.3%)		
Summer	1(1.2%)	27(29.0%)		
Autumn	29(35.4%)	30(32.3%)		
Winter	36(43.9%)	19(20.4%)		
Vomiting(times/day)			6.252	0.092
0	2(2.4%)	3(3.2%)		
1–3	48(58.5%)	39(41.9%)		
4–6	29(35.4%)	41(44.1%)		
≥7	3(3.7%)	10(10.8%)		
Diarrhea(times/day)			0.403	0.849
0	0(0.0%)	0(0.0%)		
1–3	28(34.1%)	34(36.6%)		
4–6	50(61.0%)	53(56.9%)		
≥7	4(4.9%)	6(6.5%)		
Rotavirus			11.046	0.001^*^
Negative	36(43.9%)	64(68.8%)		
Positive	46(56.1%)	29(31.2%)		
WBC (× 10^9^/L)	8.07 ± 2.55	9.06 ± 2.52	2.573	0.011^*^
NE (%)	41.25 ± 15.93	44.07 ± 18.28	1.080	0.282
LY (%)	48.71 ± 15.43	44.22 ± 16.65	−1.840	0.067
Hb (g/L)	121.16 ± 8.99	122.48 ± 13.36	0.778	0.438
PLT (× 10^9^/L)	286.68 ± 85.38	294.01 ± 86.94	0.561	0.575
AST (u/L)	46.67 ± 14.53	39.05 ± 11.74	−3.877	0.000^*^
ALT (u/L)	22.62 ± 12.86	22.62 ± 12.86	−2.370	0.018^*^
Na (mmol/L)	135.97 ± 2.97	137.90 ± 2.54	4.636	0.000^*^
Ca (mmol/L)	2.41 ± 0.14	2.43 ± 0.15	0.687	0.493
Uric acid (μmol/L)	477.77 ± 114.90	301.16 ± 90.82	−11.177	0.000^*^

*Data are N (%) or mean ± SD values*.

*SD, standard; WBC, white blood cells; NE, neutrophil; LY, lymphocyte; HB, hemoglobin; PLT, platelets; ALT, alanine aminotransferase; AST, aspartate aminotransferase; Na, serum sodium; Ca, serum calcium*.

* *Significant difference (P < 0.05) was noted between the groups*.

### Multivariate Logistic Analysis

Factors that proved significant in the univariate analysis were included in the multivariate logistic regression analysis. As shown in Table 3, rotavirus (OR = 3.042, 95% CI = 1.116–8.289, *P* = 0.030), serum sodium (OR = 0.811, 95% CI = 0.684–0.962, *P* = 0.016), and uric acid (OR = 1.018, 95% CI = 1.012–1.024, *P* = 0.000) were independent risk factors for CwG.

**Table 3:** Multivariate logistic analysis between the CwG group and the AGE group.

	**OR**	**95% CI**	***P***
Onset season			0.146
Onset season(1)	1.750	0.469–6.530	1.750
Onset season(2)	0.051	0.010–1.015	0.103
Onset season(3)	0.915	0.350–3.221	1.062
Rotavirus	3.042	1.116–8.289	0.030^*^
WBC	0.913	0.748–1.115	0.374
AST	1.035	0.988–1.083	0.146
ALT	1.013	0.974–1.054	0.520
Na	0.811	0.684–0.962	0.016^*^
Uric acid	1.018	1.012–1.024	0.000^*^

*The “ onset season” was an unordered multicategorical variable, so a dummy variable was set in the multifactorial logistic regression analysis, and “spring” was used as the criterion for comparison*.

*CI, Confidence interval; WBC, white blood cell; ALT, alanine aminotransferase; AST, aspartate aminotransferase; Na, serum sodium*.

* *Significant difference (P < 0.05) was noted between the groups*.

## Discussion

CwG was first reported as a unique clinical entity in the 1980s, and since then, related studies have been conducted around the world (1, 10, 11). To date, the clinical features and prognosis of CwG have been well reported (3, 12). The diagnosis of CwG currently relies mainly on non-specific clinical manifestations. Early diagnosis of CwG is difficult due to the lack of specific diagnostic criteria and biological indicators. However, early diagnosis of CwG is important for pediatricians to avoid excessive testing and excessive anticonvulsant treatment, and only a very few studies have explored this issue. Our study used a multifactorial study approach to explore the risk factors for CwG. As a result, rotavirus, serum sodium, and uric acid were found to be independent risk factors for CwG.

Pathogenic analysis of CwG reported that viral infections were the most common, with a small number of bacterial infections (13). A study from Korea found a norovirus positivity rate of 67.5% in children with CwG from 2014 to 2017, compared with a rotavirus detection rate of 7.5% in the same period (14). The rotavirus positivity rate in the CwG group in our study was 56.1%. Korea has a high prevalence of the rotavirus vaccine. In contrast, in China, the rotavirus vaccine is self-funded and has not been included in the national immunization program. This reason may be the main factor contributing to the significant difference. In this study, the results of the multifactorial analysis showed that rotavirus was one of the independent risk factors for CwG.

In addition to intestinal impairment, rotavirus can cause multi-system functional impairment. One of the common symptoms of neurological impairments is convulsions. Although the correlation between rotavirus and convulsions has been widely reported, the exact mechanism by which rotavirus causes benign convulsions is still unknown. By using an enzyme-linked immunosorbent assay, Yeom et al. (15) measured the IgG titer of rotavirus nonstructural protein 4 (NSP4) in the acute-phase sera of 202 children with rotavirus gastroenteritis and found a significant decrease in serum antibody levels in the convulsion group compared with the non-convulsion group. The result suggests that rotavirus infection leads to immune imbalance and that serum anti-NSP4 IgG may have a role in preventing the occurrence of rotavirus-associated benign convulsions. In addition, rotavirus infection can also lead to the abnormal release of neurotransmitters. Nitric oxide (NO), the first identified gaseous signaling molecule, is a small molecule neurotransmitter. Kawashima et al. (16) reported higher NO in serum and cerebrospinal fluid in rotavirus-infected children with CwG than in children with febrile convulsions, infectious encephalitis meningitis, and healthy children. This finding suggests the presence of NO neurotransmitter dysregulation in children with CwG. Besides the above, rotavirus infection can also lead to the excessive release of cytokines, including interleukin (IL)-8 and interleukin (IL)-10 (17). In summary, there may be multiple mechanisms by which rotavirus infection causes convulsive seizures, and more basic research is needed to clarify them in the future.

Multifactorial logistic regression analysis showed that serum sodium was an independent risk factor for CwG with an OR of 0.809, indicating that serum sodium was a protective factor for CwG. Hyponatremia is usually defined as serum sodium <135 mmol/l. Mild hyponatremia may be asymptomatic. However, the most feared complication is hyponatremic encephalopathy. Symptoms of hyponatremic encephalopathy are usually non-specific. Severe symptoms include altered consciousness, seizures, comas, myocardial ischemia, and cardiac arrhythmias (18). In children with CwG, the average serum sodium level is still in the normal range, so it is very unlikely to cause neurological symptoms and even convulsions as a direct result of cerebral edema. Similar to CwG, febrile convulsions are a common benign convulsive disorder in children. Mild hyponatremia is common in febrile convulsions. Hyponatremia is a risk factor for febrile convulsions, and the probability of recurrent febrile convulsions also increases when serum sodium levels are reduced (19, 20). In CwG, it has been shown that mild hyponatremia affects some convulsive features, especially the duration of convulsions, as children with hyponatremia suffer more prolonged convulsions than patients with normal serum sodium levels (21). Our previous study found that serum sodium levels were lower in the multiple convulsions group compared to the single convulsions group, further validating the correlation between lower serum sodium and convulsive seizures (22). Unfortunately, we still cannot clarify the causal relationship between low serum sodium and convulsions, which requires more prospective studies.

Uric acid is an end product of purine metabolism, catalyzed by the enzyme xanthine oxidase. Uric acid was previously considered an indicator for the evaluation of gout, but studies are now finding a role for uric acid in a variety of diseases. In the nervous system, uric acid has been associated with Alzheimer's disease, Parkinson's disease, and acute ischemic stroke. The regression analysis of this study showed that uric acid was an independent risk factor for CwG (OR = 1.018), as the probability of CwG increased with higher uric acid. A study from Seoul National University in Korea comparing serum levels between CwG, acute gastroenteritis, and febrile convulsions found that serum uric acid levels were higher in CwG patients even after correction for the effects of dehydration (23). Similar results have been reported in the other study (24). In animal models, local extracellular uric acid levels are significantly increased during acute borderline seizures, while controlling uric acid levels significantly reduces the number of seizures in mice (25). More basic research is needed to see if high blood uric acid plays a similar role and process in children with CwG.

This study has some limitations. Our study is a single-center retrospective study and may be subject to selection bias and recall bias. In our retrospective study, only rotavirus was routinely tested because it is the most common cause of gastroenteritis in our region. Norovirus and other pathogens were no *t test*ed. As a result, our findings may not apply to all regions, particularly those where the rotavirus vaccine is widely available. Therefore, multi-center prospective studies are needed to further evaluate the risk factors for CwG.

In conclusion, characteristics of gastroenteritis symptoms such as vomiting and diarrhea cannot be used to predict the onset of CwG. Rotavirus is an independent risk factor for CwG, while decreased serum sodium and increased uric acid can be used as early warning indicators for CwG.

## Data Availability Statement

The original contributions presented in the study are included in the article/supplementary material, further inquiries can be directed to the corresponding author/s.

## Ethics Statement

The studies involving human participants were reviewed and approved by Ethics Committee of Liyang People's Hospital. Written informed consent to participate in this study was provided by the participants' legal guardian/next of kin.

## Author Contributions

CF, WF, and CZ contributed to the study design and critically revised the manuscript for important intellectual content. CF and WF contributed to the data analysis and drafted the manuscript. CF and YY contributed to the data collection. All authors have participated sufficiently in the work to take public responsibility for the content and approved the final version of the manuscript to be published.

## Conflict of Interest

The authors declare that the research was conducted in the absence of any commercial or financial relationships that could be construed as a potential conflict of interest.

## Publisher's Note

All claims expressed in this article are solely those of the authors and do not necessarily represent those of their affiliated organizations, or those of the publisher, the editors and the reviewers. Any product that may be evaluated in this article, or claim that may be made by its manufacturer, is not guaranteed or endorsed by the publisher.

## References

[1] Castellazzi L Principi N Agostoni C Esposito S. Benign convulsions in children with mild gastroenteritis. Eur J Paediatr Neurol. (2016) 20:690–5. 10.1016/j.ejpn.2016.05.014 27292317

[2] Park SH Kim YO Kim HK Kim HS Kim BY Cheon KR. Incidence of benign convulsions with mild gastroenteritis after introduction of rotavirus vaccine. Brain Dev. (2015) 37:625–30. 10.1016/j.braindev.2014.09.002 25266417

[3] Verrotti A Moavero R Vigevano F Cantonetti L Guerra A Spezia E. Long-term follow-up in children with benign convulsions associated with gastroenteritis. Eur J Paediatr Neurol. (2014) 18:572–7. 10.1016/j.ejpn.2014.04.006 24780603

[4] Hartmann H. Benign convulsions with mild gastroenteritis-an underestimated phenomenon? Neuropediatrics. (2020) 51:313–4. 10.1055/s-0040-1715624 32818968

[5] Wu YZ Liu YH Tseng CM Tseng YH Chen TH. Comparison of clinical characteristics between febrile and afebrile seizures associated with acute gastroenteritis in childhood. Front Pediatr. (2020) 8:167. 10.3389/fped.2020.00167 32373562 PMC7176810

[6] Hao XS Liang JM Wu XM Hao YP Wang JT Liu SY. Clinical characteristics, treatment, and long-term outcomes in children suffering from benign convulsions with mild gastroenteritis: a retrospective study. BMC Pediatr. (2020) 20:516. 10.1186/s12887-020-02406-0 33172428 PMC7656682

[7] Chen B Cheng M Hong S Liao S Ma J Li T. Clinical outcome of recurrent afebrile seizures in children with benign convulsions associated with mild gastroenteritis. Seizure. (2018) 60:110–4. 10.1016/j.seizure.2018.05.020 29935410

[8] Ogawa C Kidokoro H Ishihara N Tsuji T Kurahashi H Hattori A. Splenial lesions in benign convulsions with gastroenteritis associated with rotavirus infection. Pediatr Neurol. (2020) 109:79–84. 10.1016/j.pediatrneurol.2019.05.002 32303390

[9] Lo Vecchio A Dias JA Berkley JA Boey C Cohen MB Cruchet S. Comparison of recommendations in clinical practice guidelines for acute gastroenteritis in children. J Pediatr Gastroenterol Nutr. (2016) 63:226–35. 10.1097/MPG.0000000000001133 26835905 PMC6858859

[10] Komori H Wada M Eto M Oki H Aida K Fujimoto T. Benign convulsions with mild gastroenteritis: a report of 10 recent cases detailing clinical varieties. Brain Dev. (1995) 17:334–7. 10.1016/0387-7604(95)00074-L 8579220

[11] Uemura N Okumura A Negoro T Watanabe K. Clinical features of benign convulsions with mild gastroenteritis. Brain Dev. (2002) 24:745–9. 10.1016/S0387-7604(02)00097-9 12453597

[12] Ma X Luan S Zhao Y Lv X Zhang R. Clinical characteristics and follow-up of benign convulsions with mild gastroenteritis among children. Medicine. (2019) 98:e14082. 10.1097/MD.0000000000014082 30633216 PMC6336632

[13] Chen H Zha J Zhong JM Chen Y Yu XY Xie JH. Clinical features of campylobacter-associated benign convulsions with mild gastroenteritis compared with rotavirus convulsions. Seizure. (2019) 70:20–4. 10.1016/j.seizure.2019.06.010 31238195

[14] Kim BR Choi GE Kim YO Kim MJ Song ES Woo YJ. Incidence and characteristics of norovirus-associated benign convulsions with mild gastroenteritis, in comparison with rotavirus ones. Brain Dev. (2018) 40:699–706. 10.1016/j.braindev.2018.04.001 29681427

[15] Yeom JS Kim Y-S Jun J-S Do HJ Park JS Seo J-H. NSP4 antibody levels in rotavirus gastroenteritis patients with seizures. Eur J Paediatr Neurol. (2017) 21:367–73. 10.1016/j.ejpn.2016.10.006 27847298

[16] Kawashima H Inage Y Ogihara M Kashiwagi Y Takekuma K Hoshika A. Serum and cerebrospinal fluid nitrite/nitrate levels in patients with rotavirus gastroenteritis induced convulsion. Life Sci. (2004) 74:1397–405. 10.1016/j.lfs.2003.08.014 14706570

[17] Sugata K Taniguchi K Yui A Miyake F Suga S Asano Y. Analysis of rotavirus antigenemia and extraintestinal manifestations in children with rotavirus gastroenteritis. Pediatrics. (2008) 122:392–7. 10.1542/peds.2007-2290 18676558

[18] Zieg J. Pathophysiology of hyponatremia in children [Review]. Front Pediatr. (2017) 5:8. 10.3389/fped.2017.00213 29085814 PMC5650627

[19] Baek SJ Byeon JH Eun SH Eun BL Kim GH. Risk of low serum levels of ionized magnesium in children with febrile seizure. BMC Pediatr. (2018) 18:6. 10.1186/s12887-018-1271-z 30193581 PMC6128998

[20] Kulandaivel M. Serum sodium levels and probability of recurrent febrile convulsions. Int J Sci Study. (2017) 5:5–8. 10.17354/ijss/2017/205 7641776

[21] Zifman E Alehan F Menascu S Har-Gil M Miller P Saygi S. Clinical characterization of gastroenteritis-related seizures in children: impact of fever and serum sodium levels. J Child Neurol. (2011) 26:1397–400. 10.1177/0883073811409222 21693651

[22] Fan W Fang C Yang Y Zhang C. Comparison of clinical characteristics between cluster and isolated seizures associated with benign convulsions with mild gastroenteritis. Eur J Paediatr Neurol. (2022) 36:26–9. 10.1016/j.ejpn.2021.11.008 34823071

[23] Yoo IH Kim W Cho J Kim H Lim BC Hwang H. Elevated serum uric acid in benign convulsions with mild gastroenteritis in children. J Clin Neurol. (2020) 16:181–181. 10.3988/jcn.2020.16.1.181 31942784 PMC6974815

[24] Tsujita Y Matsumoto H Nakamura Y Nonoyama S. Analysis of the blood and serum biochemistry findings in patients demonstrating convulsion with mild gastroenteritis. No to hattatsu. (2011) 43:282–4. 21800691

[25] Thyrion L Raedt R Portelli J Van Loo P Wadman WJ Glorieux G. Uric acid is released in the brain during seizure activity and increases severity of seizures in a mouse model for acute limbic seizures. Exp Neurol. (2016) 277:244–51. 10.1016/j.expneurol.2016.01.001 26774005

